# Implementation of an efficient Monte Carlo calculation for CBCT scatter correction: phantom study

**DOI:** 10.1120/jacmp.v16i4.5393

**Published:** 2015-07-08

**Authors:** Peter G.F. Watson, Ernesto Mainegra‐Hing, Nada Tomic, Jan Seuntjens

**Affiliations:** ^1^ Medical Physics Unit McGill University Montréal QC; ^2^ Ionizing Radiation Standards National Research Council of Canada Ottawa ON Canada

**Keywords:** cone‐beam CT, scatter correction, EGSnrc, Monte Carlo

## Abstract

Cone‐beam computed tomography (CBCT) images suffer from poor image quality, in a large part due to contamination from scattered X‐rays. In this work, a Monte Carlo (MC)‐based iterative scatter correction algorithm was implemented on measured phantom data acquired from a clinical on‐board CBCT scanner. An efficient EGSnrc user code (egs_cbct) was used to transport photons through an uncorrected CBCT scan of a Catphan 600 phantom. From the simulation output, the contribution from primary and scattered photons was estimated in each projection image. From these estimates, an iterative scatter correction was performed on the raw CBCT projection data. The results of the scatter correction were compared with the default vendor reconstruction. The scatter correction was found to reduce the error in CT number for selected regions of interest, while improving contrast‐to‐noise ratio (CNR) by 18%. These results demonstrate the performance of the proposed scatter correction algorithm in improving image quality for clinical CBCT images.

PACS numbers: 87.10.Rt, 87.57.Q‐

## I. INTRODUCTION

Cone‐beam computed tomography (CBCT) images suffer from poor image quality when compared to conventional fan‐beam CT, in a large part due to contamination from scattered X‐rays. X‐ray scatter manifests as streaking and cupping artifacts in CT images, as well as degraded contrast, contrast‐to‐noise ratio, and CT number accuracy.^1^, ^2^ Due to their lower than diagnostic quality, CBCT images are presently problematic for radiotherapy treatment replanning.

Current CBCT scatter correction techniques involve two main components: scatter compensation, which consists of mechanically rejecting scattered X‐rays, or compensating for them using deterministic or statistical methods;^(3)^ and scatter estimation, where the contamination from scattered X‐rays is estimated from measurements or mathematical models.^(4)^ Provided with accurate models, the most accurate method available for the estimation of scatter distributions is through a Monte Carlo (MC) simulation.^(5)^ While MC‐based CBCT scatter calculations are prohibitively long if no special techniques are used, the simulation may be accelerated by use of efficient algorithms. One approach to reduce simulation time is by reducing the number of voxels in the object volume, the number of detector pixels, and the number of simulated projection views. This approach exploits the assumption that CBCT scatter distributions are smooth and well‐behaved across each projection and between projection views. While a number of authors have published MC‐based scatter correction algorithms following this approach,^6^, ^7^, ^8^ the assumption of CBCT scatter smoothness may or may not be valid depending on the specific imaging geometry.^(9)^ To improve calculation efficiency without heavily relying on scatter smoothness assumptions, Mainegra‐Hing et al.^(9)^ employ variance reduction techniques (VRTs) and scatter projection denoising. The performance of their method has been demonstrated on idealized data,^9^, ^10^ and patient CT phantoms,^(11)^ showing an increase in efficiency of up to four orders of magnitude over an MC calculation without the use of efficiency improving techniques.^(12)^ Their software is now available as an EGSnrc^(13)^ user code, called egs_cbct.

In the original paper describing egs_cbct, Mainegra‐Hing et al.^(10)^ proposed an iterative CBCT scatter correction technique, whereby the patient geometry used to calculate the scatter distribution is updated by reconstructions from scatter‐corrected projection data obtained from the previous iteration. This procedure then converges towards the scatter‐free reconstruction. It was reported that the technique was able to correctly reproduce attenuation coefficients in mathematical phantoms. The iterative approach is interesting from a clinical implementation perspective, as it can be run directly on the raw CBCT projection data and requires no *a priori* information about the patient, such as a planning CT scan. This also eliminates any additional artifacts which may be introduced by registration errors between CT and CBCT.

In this work, we investigate the clinical feasibility of the iterative, MC‐based scatter correction of Mainegra‐Hing et al.^(10)^ by implementation on real phantom data acquired from a clinical on‐board CBCT system. The scatter correction investigated also accounts for the possible presence of an antiscatter grid in the CBCT system. Results of the scatter correction were compared with the default vendor reconstruction. To assess performance, all CBCT images were compared with the clinical benchmark of a planning CT scan.

## II. MATERIALS AND METHODS

### A. Scatter correction definitions

If $S_{i}$ is the scatter contribution to the signal in pixel i for a given projection, and $P_{i}$ is the primary contribution, the total pixel signal will be $R_{i}=P_{i}+S_{i}$. The quantity reconstructed by the CBCT scanner is

(1) $$r_{i}=\text{ln}\frac{B_{i}}{R_{i}},$$

where $B_{i}$ is the pixel signal from a blank (air) scan. We can then define the scatter‐free quantity

(2) $$p_{i}=\text{ln}\frac{B_{i}}{P_{i}}=\text{ln}\frac{B_{i}}{R_{i}-S_{i}}.$$

We then assume that the major differences between measured and simulated pixel intensity are due to differences in simulation attenuation properties; that is the ratio between measured and simulated pixel signal (both primary and scatter) is a constant. $P_{i}$ can be replaced with $\tilde{P}\times R_{i}/\tilde{R_{i}}$, where symbols with a tilde denote MC‐derived quantities. Equation (2) then becomes

(3) $$p_{i}=r_{i}-\text{ln}\frac{{\tilde{P}}_{i}}{{\tilde{P}}_{i}+{\tilde{S}}_{i}}.$$

To compensate for errors introduced by this assumption, a relaxation term of $\alpha (r_{i}-{\tilde{r}}_{i})$ is added, with α left as a free parameter. To account for the presence of an antiscatter grid, the simulated scatter intensity is modulated by a grid parameter (β). The scatter‐free quantity in its final form is then given by

(4) $$p_{i}=(1+\alpha )r_{i}-\alpha {\tilde{r}}_{i}-\text{ln}\frac{{\tilde{P}}_{i}}{{\tilde{P}}_{i}+\beta {\tilde{S}}_{i}}.$$

This expression is an extension to the proposed approach of Mainegra‐Hing et al.^(10)^

It can be shown that β is equivalent to the reduction of scatter‐to‐primary ratio (SPR) due to an antiscatter grid by looking at the quantity in the logarithm of Eq. (4),

(5) $$\frac{{\tilde{P}}_{i}}{{\tilde{P}}_{i}+\beta {\tilde{S}}_{i}}=\frac{1}{1+\beta ({\tilde{S}}_{i}/{\tilde{P}}_{i})},$$

where $\tilde{S}/\tilde{P}$ is the SPR simulated without an antiscatter grid. We see that β can then be expressed as:

(6) $$\beta =\frac{SPR_{grid}}{SPR_{no-grid}}.$$

For a given configuration, β can be found in literature. Sisniega et al.^(14)^ report that the reduction in SPR achieved with a 10:1 antiscatter grid for a CBCT linac configuration (SAD=100 cm,SDD=150 cm) is in the neighborhood of 0.5.

### B. Iterative scatter correction algorithm

The scatter correction used in this work follows the iterative algorithm proposed by Mainegra‐Hing et al.^(10)^ To initialize the algorithm, raw projection data from the to‐be‐corrected CBCT scan must be acquired. A 3D reconstruction is performed from this projection data to yield the raw, uncorrected CBCT slices.

Once this 0th iteration has been performed, the steps of the scatter correction algorithm are as follows:

1. Convert reconstructed voxel attenuation coefficients (μ) to material densities.
2. Import density phantom into MC simulation, and compute primary and scatter distributions for each projection angle.
3. Apply scatter correction Eq. (4) to raw projection pixel data.
4. Perform 3D reconstruction from projection data
5. Go to step 1.

**Figure 1:**
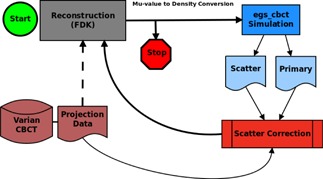
Flowchart of iterative scatter correction algorithm.

The iterative procedure stops at step 4 when a convergence criterion has been reached. A flowchart depicting the iterative correction algorithm is shown in Fig. 1.

### C. Image reconstruction

After acquiring a CBCT scan, the raw projection data was extracted from the OBI reconstructor computer. This data was normalized to the X‐ray tube output chamber, then smoothed with a median filter. Air normalized projection values were then calculated and input into an FDK reconstruction algorithm,^(15)^ using a Shepp‐Logan convolution filter.^(16)^ Partial arc reconstruction was handled by weighting overlapping projections with a smooth sinogram window.^(17)^

### D. MC simulation

The CBCT simulation was performed using egs_cbct, an EGSnrc user code. The detector signal was estimated by scoring air kerma using forced detection, where contributions from photons aimed at the detector are scored before crossing the plane, accounting for the attenuation through the geometry. Photons were simulated down to 10 keV and electron transport was turned off. Material photon cross sections were calculated from the XCOM database.^(18)^ Compton interactions were modeled in the relativistic impulse approximation taking into account binding and Doppler broadening.^(13)^ Coherent scattering (Rayleigh) was simulated using the independent atom approximation.

To further improve the calculation efficiency of CBCT scatter estimation, egs_cbct makes use of a number of variance reduction techniques (VRTs).^(9)^ To enhance the number of interactions occurring deep in the phantom where most of the scatter signal originates, a photon path length biasing technique was implemented which does not depend on the photon direction. A combination of fixed interaction splitting and Russian Roulette (RR) was used to increase the number of photons directed towards the scoring plane. In this splitting + RR scheme, after its first interaction, a photon is split into $N_{p}$ photons with statistical weight $1/N^{p}$. To reduce the time wasted transporting photons aimed away from the scoring plane, RR is used to “kill” these photons with a probability of $1‐1/N_{s}$. A split photon surviving the RR will have a weight of $N_{s}/N_{p}$. This photon can then interact and be split $N_{s}$ times, with the same RR procedure applied. This approach ensures that all photons reaching the detector have the same statistical weight ($1/N_{p}$). To reduce the amount of time spent transporting photons not aimed at the detector through heterogeneous regions, delta transport, or Woodcock tracing, was employed.^(19)^ Delta transport is a technique whereby photons are allowed the possibility to undergo a fictitious interaction. This fictitious interaction leaves the energy and direction of the photon unaltered, and has a cross section equal to the difference of the maximum cross section in the volume and the voxel cross section (i.e., voxels with a large cross section will have a small fictitious interaction cross section, and vice versa). This approach gives the entire geometry a homogeneous total (real + fictitious) photon cross section, allowing the photon to be transported directly to the interaction site, eliminating the need for tedious ray tracing. A locally adaptive smoothing algorithm^(20)^ was applied to the scatter distributions to further decrease simulation time.

### E. CT‐to‐density conversion

To transport particles through a CT‐acquired geometry using Monte Carlo, each voxel of the CT image must be associated to a material type (atomic number Z and atomic mass number A)^*^ and mass density (ρ). For each CBCT‐scanned object, a ramp file was created containing a list of relevant materials, their nominal attenuation coefficient (μ_mat_), and mass density ($\rho_{\text{mat}}$). Once assigned to a material, voxel mass density was calculated by a fractional method, where for a voxel with $\mu_{i}$, density was given by

(7) $$\rho_{i}=\frac{\mu_{i}}{\mu_{mat}}\rho_{mat}.$$

In this way, the ratio of $\mu_{i}$ to $\mu_{\text{mat}}$ is recovered in the simulation by dividing $\rho_{i}$ by $\rho_{\text{mat}}$. Table 1 lists an example of μ ranges used in this work to assign materials to voxels reconstructed from a scan of a Catphan 600 phantom (The Phantom Laboratory, Salem, NY).

**Table 1:** Attenuation coefficient ranges used for material assignment and density conversion for a Catphan 600 phantom. $\mu_{\text{mat}}$ was determined from an EGSnrc simulation for the CBCT X‐ray source spectrum

μ (1/cm)	*Material*	$\mu_{\text{mat}}\;(1/\text{cm})$	$\rho_{\text{mat}}\;(g/{\text{cm}}^{3})$
<2.5E‐4	Vacuum	0.0	0.0
2.5E‐4 to 0.09	Air	5.18E‐4	1.205E‐3
0.09 to 0.189	PMP^a^	0.178	0.83
0.189 to 0.209	LDPE^b^	0.200	0.93
0.209 to 0.23	Polystyrene	0.217	1.06
0.23 to 0.251	Water	0.243	1.00
0.251 to 0.287	Acrylic	0.259	1.19
0.287 to 0.4	Delrin	0.315	1.41
>0.4	Teflon	0.485	2.10

a ^a^ Polymethylpentene

b ^b^ Low‐density polyethylene

### F. Algorithm implementation on VMS OBI system

All CBCT images in this study were acquired using a VMS (Varian Medical Systems, Palo Alto, CA) On‐Board Imager (OBI) system, mounted on a VMS Novalis medical linear accelerator. The system consisted of a kV X‐ray source (VMS G242) and flat‐panel detector (PaxScan 4030CB) whose beam central axis was orthogonal to the treatment beam central axis. The flat‐panel detector had dimensions of 39.7 by 29.8 cm (1024 by 768 pixels), and was equipped with a focused 10:1 antiscatter grid. The source‐to‐axis (SAD) distance of the system in question was 100 cm, and the source‐to‐detector distance (SDD) was 149.88 cm. In this work, the OBI system was operated using the Standard Dose Head scanning protocol (100 kVp and 145 mAs), with the bowtie compensator removed. In this protocol, 372 projection images were acquired over a 200° partial arc scan.

The iterative scatter correction was applied to a CBCT scan of a Catphan 600 phantom. The configuration of the egs_cbct simulation was made to resemble the OBI system operated using the Standard Dose Head protocol with the bowtie filter removed. The X‐ray spectrum was based on a model of a COMET MXR‐320 tube operated at 100 kVp, and includes inherent filtration and attenuation through its beryllium window ($E_{\text{eff}}=45\;\text{keV}$). Beam intensity was assumed to be constant across solid angle, and was collimated to the projected dimensions of the flat panel detector. The detector was assumed to have an ideal energy response, and scoring plane resolution was set to 256 by 192 pixels of 1.552 by 1.552 mm^2^. This coarse scoring helps to increase computational efficiency by taking advantage of the relatively low spatial frequency of scatter distributions.^(21)^ Once the egs_cbct simulation was complete, the simulated projection images were upsampled using bicubic interpolation to match the resolution of the OBI system (1024 by 768 pixels).

The CBCT projection data was reconstructed to have a size of 512 by 512 pixels, with 0.51 by 0.51 mm^2^ per pixel. Slice thickness in the longitudinal direction was 2.7 mm. Prior to converting the reconstructed voxel attenuation coefficients to densities as part of the scatter correction, the reconstructed slices were downsampled to a resolution of 256 by 256. This downsampling helps to increase the simulation efficiency by reducing the number of voxel boundaries encountered during particle transport.

### G. Comparison with fan‐beam CT and evaluation

In addition to the CBCT scan, the Catphan was also scanned on a fan‐beam CT (Philips Brilliance Big‐Bore CT (Philips Healthcare, Andover, MA)) used for patient treatment planning at the Montreal General Hospital. The planning CT (pCT) images were acquired axially at 120 kVp, 200 mAs, with 3 mm slice thickness (3 mm collimation). This allowed for a comparison of the scatter corrected CBCT images with “clinical ground truth” CT data. A CBCT CT number normalization was performed by setting the Hounsfield units (HU) for Catphan water‐equivalent material to agree between the scatter corrected CBCT scan and the planning CT.

To quantify the reconstructed CT number accuracy, a region of interest (ROI) was selected for each of the eight Catphan embedded contrast materials. In each ROI, the mean CT number and standard deviation was recorded. The overall CBCT image CT number error was estimated by the square root of the mean square error (RMSE):

(8) $$RMSE=\sqrt{\bar{{\left(\mu_{i}-\mu_{i,ref}\right)}^{2}},}$$

where $\mu_{t}$ and $\mu_{i,\text{ref}}$ are the mean values of the CBCT and planning CT ROI for material *i*, respectively. Image quality was also quantified by the contrast‐to‐noise ratio (CNR), defined as

(9) $$CNR=\frac{\left|\mu_{A}-\mu_{B}\right|}{\sqrt{\sigma_{A}^{2}+\sigma_{B}^{2}}},$$

where $\mu_{A}$ and $\mu_{B}$ are the mean values, and $\sigma_{A}$ and $\sigma_{B}$ are the standard deviations of CT numbers in two neighboring ROIs.

## III. RESULTS & DISCUSSION

### A. Mathematical water phantom

**Figure 2:**
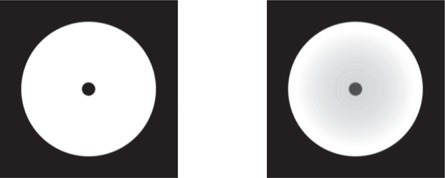
Mathematical water phantom with air cavity used in the study, reconstructed from (left) primary photons only, and (right) primary + scattered photons.

The performance of the iterative scatter correction was first assessed on a mathematical phantom, using a monoenergetic 60 keV X‐ray source. The phantom consisted of a homogeneous water cylinder (20 cm diameter by 20 cm length) containing a central cylindrical air cavity (2 cm diameter by 10 cm length). Exploiting the phantom symmetry, only the 0° projection was computed. This projection was then duplicated to obtain the complete scan (360 projections over 360°). Figure 2 shows the simulated water phantom scan when reconstructed from primary photons only (left panel), and (right panel) primary + scattered photons. Here, a cupping artifact and an air cavity CT number inaccuracy are quite noticeable in the primary + scatter scan. The scatter correction was applied with β=1, as no antiscatter grid was present in the simulation. Two relaxation parameters, α=0 and α=0.5, were investigated.

**Figure 3:**
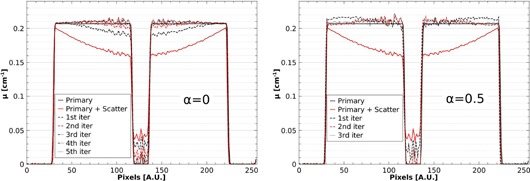
Profiles of the reconstructed water phantom after iterative scatter correction using relaxation parameters of (left) α=0 and (right) α=0.5. It can be seen that the correction reduces a scatter‐induced cupping artifact and air cavity CT number error.

**Figure 4:**
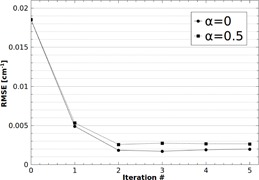
RMSE in the central slice of the mathematical water phantom as a function of scatter correction iteration number. After only two iterations, the RMSE for both cases of α can be seen to converge towards a minimum value.

Figure 3 shows profiles through the central horizontal line of the reconstructed water phantom for both primary only and primary + scatter scans (Fig. 2), and the results after each iteration of the scatter correction. For both chosen values of α, after each iteration the reconstructed attenuation coefficient values appear to converge towards the scatter‐free result. This convergence can be quantified by calculating the RMSE of the reconstructed voxels for each iteration, using the scatter‐free scan as reference. Figure 4 shows the RMSE in the central slice as a function of scatter correction iteration number, starting with the uncorrected scatter + primary scan (0th iteration). After only two iterations, the RMSE for both cases of α can be seen to converge towards a minimum value. This residual error is largely due to statistical noise and correlations introduced by reusing the 0° projection for all projection angles (visible as fluctuations near the center of the phantom profiles in Fig. 3). It was found that using a nonzero α has negligible improvement on convergence rate, while enhancing voxel errors.

### B. Catphan 600 phantom on VMS OBI system

**Figure 5:**
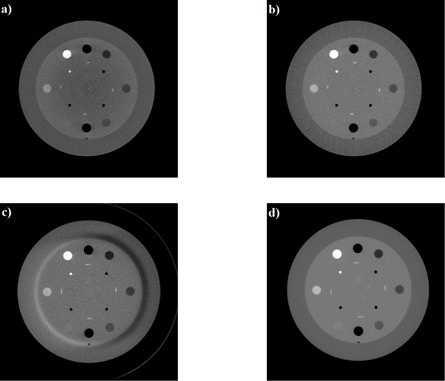
Reconstructed Catphan images from: a) CBCT without scatter correction; b) CBCT with scatter correction; c) Varian default algorithm, and d) fan‐beam planning CT (reference).

**Figure 6:**
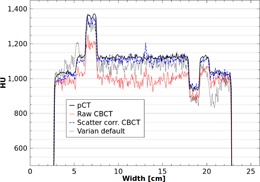
Profiles across the central horizontal line of the Catphan images in Fig. 5.

Next, the iterative scatter correction was performed on measured phantom data, using α=0 and β=0.5. The results were found to converge visibly after three iterations of the correction. The performance of the scatter correction is evident in the reconstructed Catphan images, as shown in Fig. 5. When reconstructed from uncorrected projection data, the CBCT image has an obvious cupping artifact and large CT number errors, as shown in Fig. 5(a). Implementation of the proposed scatter correction significantly reduces these scatter artifacts (Fig. 5(b)). As another test of performance, the scatter corrected images were compared with the images provided by the Varian OBI default algorithm for the same scan protocol (Fig. 5(c)). Qualitatively, the default images were found to have more noise than the scatter corrected images, and contained a crescent artifact due to the presence of the bowtie filter. A comparison of profiles across the central horizontal line in these images is shown in Fig. 6. In both figures (Fig. 5(d) and Fig. 6), the fan‐beam planning CT image is shown for reference.

As described in the Materials & Methods section G, a quantitative analysis of image quality was investigated. An ROI was selected for each of the eight Catphan contrast materials (see Fig. 5). Each ROI was drawn as a circular region of size 225 voxels, centered on the contrast material, with care taken to avoid the material edge. The mean CT number for each ROI from the CBCT images was then compared with the mean value obtained in the fan‐beam planning CT images, and the CT number difference (ΔHU) was calculated. The CNR for each contrast material was computed, referenced to the surrounding background phantom medium in which the materials were embedded. Table 2 summarizes these results.

In nearly all materials, the CBCT CT number accuracy was greatly improved after scatter correction. The only exception was for PMP, whose ΔHU, in absolute terms, was left relatively unchanged (ΔHU=−19 for raw CBCT versus ΔHU=24 after scatter correction), with the uncorrected HU the closest to the pCT HU. While the CT number accuracy of the air cavities was improved, they had the poorest performance when compared to the other material inserts. This overestimation of air HU is most likely due to beam hardening, which is not accounted for in the scatter correction algorithm. Compare this to the Varian default algorithm, which applies a variety of corrections and calibrations, and had a relatively consistent ΔHU for all materials.

The CBCT image CT number error as estimated by the RMSE is shown in Table 3, along with the average standard deviation (SD) and CNR for all ROIs. There is improvement in the overall CT number accuracy after applying the proposed scatter correction, as witnessed by the reduction of RMSE from 175.8 to 65.3 HU. The Varian default algorithm had the lowest CT number error, at 47.7 HU. However, if the air ROIs are ignored in the calculation of RMSE, the scatter corrected result improves to 34.2 HU, while the Varian default degrades slightly to 50.4 HU. This highlights that the CT number inaccuracy of air is a dominant contribution to the RMSE of the scatter corrected image, and could be improved further by introducing a beam hardening correction to the scatter correction procedure.

To put these values into perspective, it is worth mentioning that a perfect agreement between planning CT and CBCT CT numbers (i.e., RMSE=0 HU) is not expected. Due to differences in beam quality and other scanner dependent features, interscanner CT numbers can easily differ by more than 10 HU.^22^, ^23^, ^24^ In the context of dose calculations, it has been shown that MV photon beam dose distributions are relatively insensitive to CT number errors.^(25)^ Poludniowski et al.^(25)^ reported that CT number errors of up to 50 HU resulted in calculated dose discrepancies of less than 3%.

**Table 2:** Reconstructed mean CT number, standard deviation (SD), and CNR for Catphan material ROIs. Absolute differences (ΔHU) from fan‐beam planning CT values are shown for the CBCT images. The CT number scale used here defines water as 1024 HU

		*Air 1*	*Air 2*	*PMP*	*LDPE*	*Polysty*.	*Acrylic*	*Delrin*	*Teflon*
Mean HU	pCT	68±11	68±11	853±7	940±7	994±7	1145±9	1363±9	1936±10
	Raw CBCT	302±21	294±23	834±16	901±16	939±17	1043±19	1199±17	1621±21
	Scat. Corr.	185±22	184±31	877±17	958±17	1005±18	1119±17	1327±18	1872±20
	Varian default	27±6	32±14	785±30	881±34	940±32	1105±33	1321±41	1965±37
ΔHU	Raw CBCT	234	226	−19	−39	−55	−102	−164	−315
	Scat. Corr.	117	116	24	18	11	−26	−36	−64
	Varian default	−41	−36	−68	−59	−54	−40	−42	29
CNR	pCT	80.7	80.7	27.0	16.9	12.7	2.2	21.3	66.8
	Raw CBCT	26.7	25.8	8.3	5.5	3.8	0.6	7.1	21.7
	Scat. Corr.	33.9	26.2	9.9	6.6	4.4	0.2	8.8	28.8
	Varian default	34.2	31.4	6.8	4.2	3.0	0.7	4.9	18.7

**Table 3:** The RMSE, averaged standard deviation and CNR for material ROIs of the Catphan phantom

	*RMSE (HU)*	*SD (HU)*	*CNR*
pCT	‐	9.0	38.5
Raw CBCT	175.8	18.7	12.5
Scat. Corr.	65.3	20.2	14.8
Varian default	47.7	28.4	13.0

Improved CNR is arguably more important than CT number errors regarding the use of CBCT images in treatment planning, as it allows for better delineation of tissues and structures.^(26)^ The scatter correction was found to enhance the average image CNR, increasing it from 12.5 to 14.8, representing an improvement of 18%. The CNR of the Varian default algorithm was only marginally better than the raw CBCT (13.0, or 4% improvement), mainly due to image noise.

### C. Calculation time

For each simulated projection angle, $∼10^{7}$ particle histories were run. This yielded an average statistical uncertainty of 2% for primary projections, 4.2% (presmoothing) and 0.3% (postsmoothing) for scatter projections. Running on a single core of a 2.66 GHz Intel Xeon processor, the simulation of one projection angle required 35 min. The FDK reconstructions were completed in less than 10 min. Using a computer cluster of 80 CPU cores, one complete iteration of the scatter correction could be obtained in 3 hrs.

There are options available to further decrease the simulation time. Instead of an MC simulation, the primary photon signal could be estimated by an efficient ray tracing algorithm, essentially eliminating primary statistical uncertainty.^(11)^ For scatter projection simulation, the parameters of the VRTs and smoothing algorithm used by egscbct could be optimized for the specific geometry.^(12)^ Provided no bias was introduced, scatter scoring could be made even coarser, and fewer projection angles could be simulated. The statistical uncertainty of the smoothed scatter distributions in this work was 0.3%; however, a more relaxed uncertainty criterion may be acceptable for scatter correction. Allowing for a larger scatter statistical uncertainty would reduce the required number of simulated photon histories, reducing computation time. After implementation of these suggestions, we believe a decrease in overall computation time of two orders of magnitude is possible.

For clinical feasibility, it would be beneficial to have implementation on a desktop computer, rather than relying on access to a computer cluster. Multicore desktop workstations of up to 36 cores are now commercially available. Implementation of egscbct on a graphics processing unit (GPU)^(27)^ is another potential option for desktop parallelization.

## IV. CONCLUSIONS

This paper demonstrates the feasibility of implementing an MC‐based iterative scatter correction on Catphan 600 phantom images acquired from a clinical on‐board CBCT scanner. The scatter correction requires no *a priori* patient information, and is run directly on raw CBCT projection data. Primary and scatter distributions were calculated using egs_cbct, an EGSnrc user code shown to increase efficiency by up to four orders of magnitude compared to an analog simulation. The scatter correction has been shown to be successful in reducing scatter‐based image artifacts, such as cupping and CT number inaccuracies, while improving CNR by 18%.

Prior to clinical implementation on patient data, the inclusion of a bowtie compensator, accurate X‐ray source spectrum, detector energy response, and beam hardening should be considered as part of the scatter correction. These effects are not expected to significantly increase simulation time. The X‐ray source simulation with bowtie compensator can be performed in a separate simulation, yielding a phase space file at the exit plane of the compensator containing photon energies, positions, and directions. This phase space file could then be used as the photon source in the egs_cbct simulation. Including the bowtie compensator may even help to decrease simulation time by reducing the amount of patient scatter.^(28)^ Detector response could be calculated quickly by using a lookup table that maps the detector response to a photon's energy and direction. With accurate knowledge of the X‐ray spectrum leaving the bowtie compensator, a higher‐order beam hardening correction could be incorporated into the scatter correction algorithm.

With further improvements to simulation efficiency through ray tracing, optimization of VRTs, and GPU implementation, this work paves the way for a clinical MC‐based, patient‐specific CBCT scatter correction. These are promising results towards reliable use of CBCT images in adaptive treatment replanning.

## ACKNOWLEDGMENTS

We would like to thank Peter Munro (Varian Medical Systems) for his assistance on working with OBI raw data, Glenn Wells (Ottawa Heart Institute) for providing the FDK reconstruction code, and Rebecca Fahrig (Stanford University) for her advice on image reconstruction. This work was partially supported by grants from the Canadian Institutes of Health Research, CIHR MOP 102550, and Natural Sciences and Engineering Research Council Discovery grants number 298191. Also, this work was partially supported by the Medical Physics Research Training Network, Natural Sciences and Engineering Research Council/Collaborative Research and Training Experience initiative 432290. The RI of the MUHC is gratefully acknowledged for partial scholarship support.
